# Supplementary material for: Ropinirole Cotreatment Prevents Perivascular Glial Recruitment in a Rat Model of L-DOPA-Induced Dyskinesia
Source: Cells. 2023 Jul 14;12(14):1859. doi: 10.3390/cells12141859 (PMC10378233; doi:10.3390/cells12141859)
Supplement: Supplementary file 1 [file cells-12-01859-s001.zip › cells-2398832-supplementary.pdf]

Supplemental Material

Ropinirole Cotreatment Prevents Perivascular Glial Recruitment in a Rat Model of L-DOPA-Induced Dyskinesia

Osama F. Elabi †, Elena Espa *,†, Katrine Skovgård, Silvia Fanni and Maria Angela Cenci *

Basal Ganglia Pathophysiology Unit, Department of Experimental Medical Science, Lund University, 221 84 Lund, Sweden
* Correspondence: elena.espa@med.lu.se (E.E.); angela.cenci_nilsson@med.lu.se (M.A.C.)
† These authors contributed equally to this work.

1. Tyrosine Hydroxyles immunostaining after unilateral 6-OHDA lesions

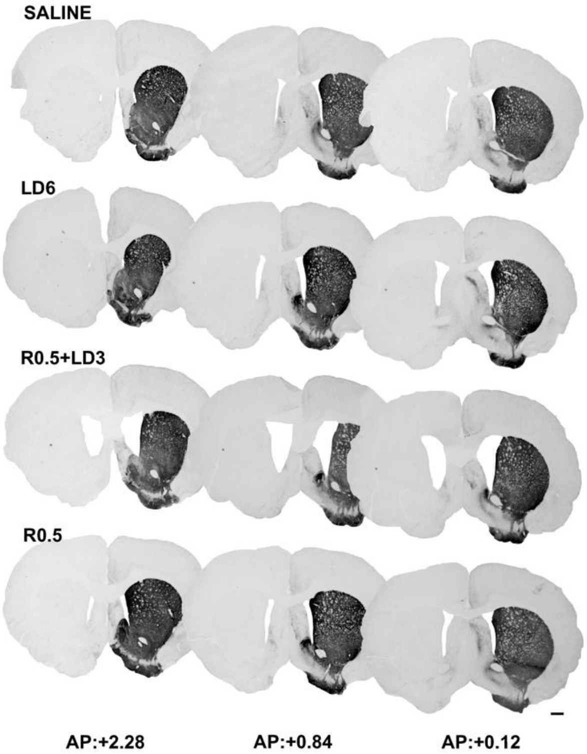

Figure S1. Overview of striatal brain sections immunostained for Tyrosine Hydroxylase (TH) shows the typical MFB lesion-induced pattern of severe DA fibre loss on the side ipsilateral to the lesion. Pictures were taken from animals treated with saline, LD6, R0.5+LD3 and R0.5 throughout three different rostrocaudal levels according to [1]. Scale bar: 1000µm.

2. Axial, limb and orolingual dyskinesia subtypes

For a comprehensive and detailed protocol on dyskinesia ratings, we referred to [2].

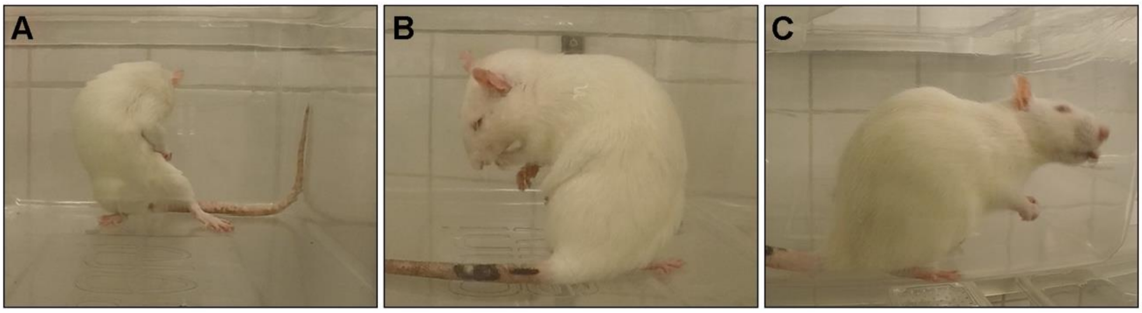

Figure S2. Sample of screenshots taken from video-recorded sessions of dyskinesia under L-dopa mono treatment and L-DOPA-ropinirole cotreatment. **A,** Axial; **B**, forelimb and **C**, orolingual dyskinesia.

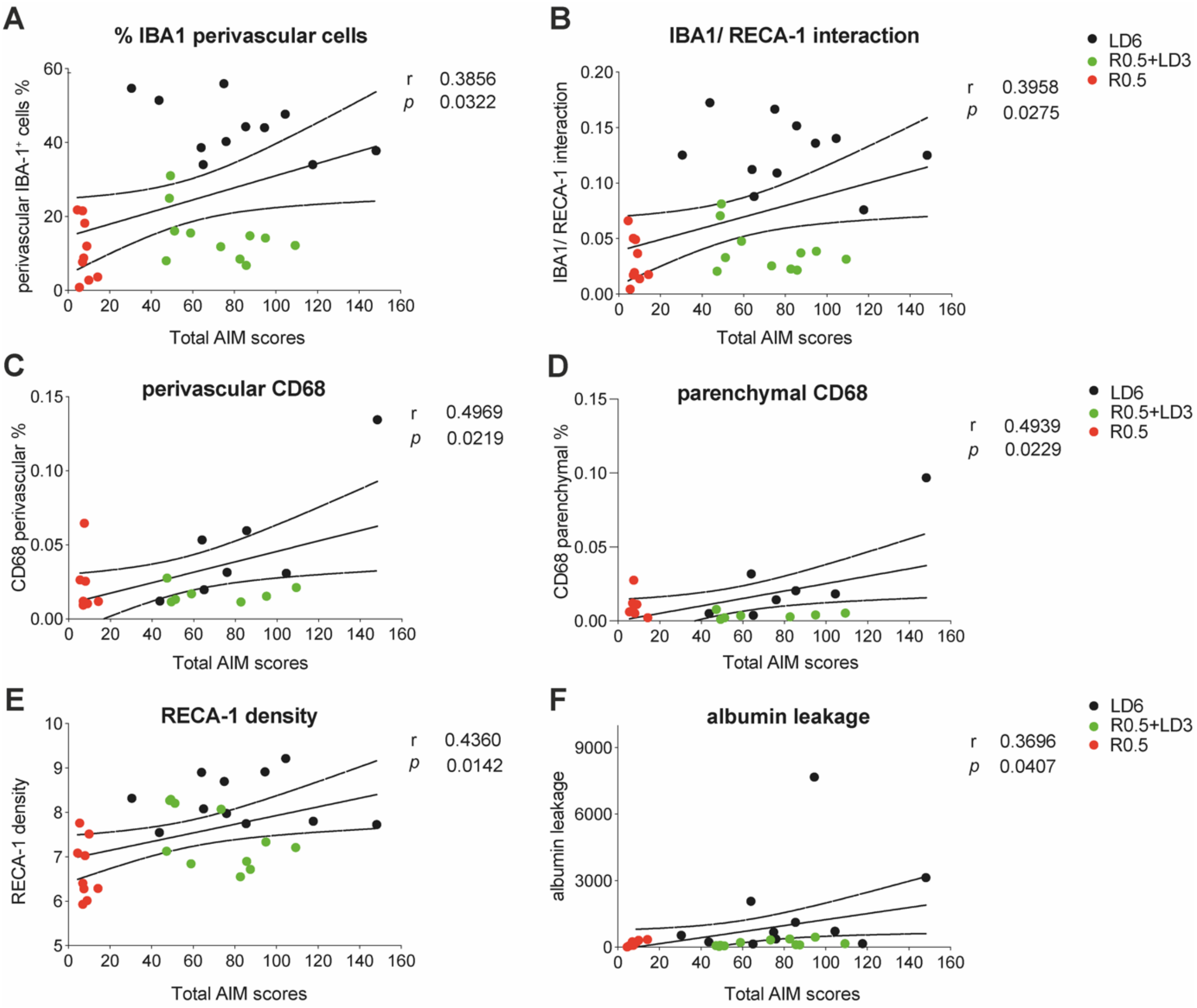

Figure S3. Correlation analysis between AIMs scores and histological analysis for L-DOPA (LD6), R0.5 and L-DOPA cotreatment (R0.5+LD3) and ropinirole (R0.5) groups. Pearson's correlation r and p-value are shown for each graph. **A:** Percentage of perivascular IBA-1 cells; **B:** IBA-/RECA-1 interaction; **C:** perivascular CD68; **D:** parenchymal CD68; **E:** RECA-1 density and **F:** albumin leakage.

References

1. Paxinos, G.; Watson, C. *The rat brain in stereotaxic coordinates*, 6th ed.; Academic Press/Elsevier: Amsterdam ; Boston 2007.

2. Cenci, M.A.; Lundblad, M. Ratings of L-DOPA-induced dyskinesia in the unilateral 6-OHDA lesion model of Parkinson's disease in rats and mice. *Curr Protoc Neurosci* **2007**, *Chapter 9*, Unit 9 25, doi:10.1002/0471142301.ns0925s41.
